# Formulation and Evaluation of *Helichrysum italicum* Essential Oil-Based Topical Formulations for Wound Healing in Diabetic Rats

**DOI:** 10.3390/ph14080813

**Published:** 2021-08-19

**Authors:** Marijana Andjić, Biljana Božin, Nevena Draginić, Aleksandar Kočović, Jovana N. Jeremić, Marina Tomović, Andjela Milojević Šamanović, Nebojša Kladar, Ivan Čapo, Vladimir Jakovljević, Jovana V. Bradić

**Affiliations:** 1 Department of Pharmacy, Faculty of Medical Sciences, University of Kragujevac, 34000 Kragujevac, Serbia; andjicmarijana10@gmail.com (M.A.); nevenasdraginic@gmail.com (N.D.); salekkg91@gmail.com (A.K.); marinapop@gmail.com (M.T.); jovanabradickg@gmail.com (J.V.B.); 2 Department of Pharmacy, Faculty of Medicine, University of Novi Sad, 21000 Novi Sad, Serbia; biljana.bozin@mf.uns.ac.rs (B.B.); nebojsa.kladar@mf.uns.ac.rs (N.K.); 3 Center for Medical and Pharmaceutical Investigations and Quality Control, University of Novi Sad, 21000 Novi Sad, Serbia; ivan.capo@mf.uns.ac.rs; 4 Department of Dentristy, Faculty of Medical Sciences, University of Kragujevac, 34000 Kragujevac, Serbia; andjela-kg@hotmail.com; 5 Department of Histology and Embryology, Faculty of Medicine, University of Novi Sad, 21000 Novi Sad, Serbia; 6 Department of Physiology, Faculty of Medical Sciences, University of Kragujevac, 34000 Kragujevac, Serbia; drvladakgbg@yahoo.com; 7 Department of Human Pathology, 1st Moscow State Medical, University IM Sechenov, 119991 Moscow, Russia

**Keywords:** *Helichrysum italicum* essential oil, wound healing, ointment, gel, oxidative stress

## Abstract

As proper wound management is crucial to reducing morbidity and improving quality of life, this study evaluated for the first time the wound healing potential of *H. italicum* essential oil (HIEO) prepared in the form of ointment and gel in streptozotocin-induced diabetic wound models in rats. After creating full-thickness cutaneous wounds, forty-eight diabetic rats were divided into six groups: (1) negative control; (2) positive control; (3) ointment base; (4) gel base; (5) 0.5% HIEO ointment (6) 0.5% HIEO gel. Wound healing potential was determined by the percentage of wound contraction, hydroxyproline content, redox status, and histological observation. A significant decrease in the wound size was observed in animals treated with HIEO formulations compared with other groups. The HIEO groups also showed a higher level of total hydroxyproline content, and more pronounced restitution of adnexal structures with only the underlying muscle defect indicating the incision site. Hence, our results legitimate the traditional data of the pro-healing effect of HIEO because HIEO in both formulations such as gel and ointment exhibited the significant wound repairing effect in the incision wound model.

## 1. Introduction

Diabetes mellitus (DM) represents a group of chronic, metabolic diseases, with the main feature of chronic hyperglycemia caused by defects in insulin secretion, insulin efficacy, or, most often, both [1]. According to International Diabetes Federation, DM, as the largest global epidemic of the twenty-first century, affects more than 420 million individuals with constantly increasing prevalence [2]. DM is associated with a high risk of developing serious micro- and macrovascular complications. One of the most serious complications is impaired wound healing, which often leads to the development of chronic wounds and amputations [3]. Wound healing is delayed due to disturbance in each phase of wound healing, i.e., the hemostasis, inflammation, proliferation, and remodeling phases [4]. The altered inflammatory response, decreased collagen content, and oxidative stress also significantly contribute to poor wound healing in patients with DM [3,5]. Worldwide, 20% of diabetic patients suffer from diabetic foot ulcers, known as impaired, persistent, and non-healing wounds, which are linked to lower-extremity amputation both in the final stage of DM and in newly diagnosed patients [3,6].

Diabetes foot ulcers and a high risk of amputation and mortality can impact patients’ quality of life, life roles, and body image, as well as the financial burden placed on patients and their families [7]. Therefore, proper wound management is crucial for reducing morbidity and improving quality of life. Nowadays, there are many synthetic drugs used for wound treatment; however, their application is associated with the occurrence of allergies, irritations, and other skin complications. Therefore, herbal products have been in the focus of scientific research in recent years due to their great potential efficacy and better safety profile.

*Helichrysum. italicum* (Roth) G. Don. (*H. italicum*) is a typical Mediterranean plant belonging to the *Asteraceae* family, called immortelle, curry plant or sandy everlasting because of bright yellow-colored inflorescences that do not wither [8,9]. It possesses a wide range of biological activities, such as antimicrobial, anti-inflammatory and antioxidant properties and traditional use is related to respiratory, digestive and skin inflammatory conditions [10,11]. The important role of *H. italicum* is reflected in the use of its essential oil in the perfume industry and aromatherapy. In traditional medicine it is widely used in the treatment of wounds and skin conditions such as hematoma and scars. Phytochemical investigations suggest the presence of terpenes such as α-pinene, neryl-acetate, nerol, α- and γ-curcumin, as well as geranyl-acetate in *H. italicum* essential oil (HIEO). However, the chemical composition varies in relation to the geographic origin, vegetation cycle and whether fresh or dried plant material was used [12,13]. To the best of our knowledge, there are no studies revealing the wound healing potential of *H. italicum* essential oil, and the underlying mechanisms have not been reported so far. Moreover, the potential difference in the efficacy of *H. italicum* essential oil incorporated in different semi-solid dosage forms has not been clarified yet. Therefore, the aim of the present study was to evaluate and compare the wound healing potential of essential oil of *H. italicum* prepared in form of ointment and gel in a diabetic rat model.

## 2. Results

### 2.1. GC-MS Analysis of H. italicum Essential Oil

The chemical composition of the *H. italicum* essential oil determined by GC/MS analysis is shown in Table 1. Forty-six components identified in the *H. italicum* essential oil represent 98.37% of the total essential oil composition, and many of these compounds are present in trace amounts. Sesquiterpene hydrocarbons, constituted with a total relative content of almost 60%, were the most abundant class of chemical compounds, while the monoterpene hydrocarbons constituted 18.52% of the total composition. The major components detected were γ-Curcumene (14.07%), Neryl acetate (12.96%), α-Pinene (12.38%), β-Selinene (11.27%) and α-Selinene (7.27%).

### 2.2. Antioxidant Activity

The antioxidant activity of *H. italicum* essential oil was appraised by employing five test systems: 2,2-diphenyl-1-picrylhydrazyl (DPPH·) free radical scavenging assay, hydroxyl ion (·OH), nitric oxide (·NO), lipid peroxidation (LP) and ferric reduction antioxidant potential (FRAP) test (Table 2). Essential oil of *H. italicum* exhibited scavenging of DPPH radical with IC_50_ value of 4.45 ± 0.44 μg/mL. PG showed better radical scavenging activity in DPPH assay with an IC_50_ value of 0.69 ± 0.03. Hydroxyl ion scavenging assay showed best results for BHT (0.03 ± 0.01 μg/mL) followed by ascorbic acid (2.03 ± 0.39 μg/mL, while propyl gallate and essential oil showed comparatively higher IC_50_ values (9.01 ± 0.48 μg/mL and 13.33 ± 1.11 μg/mL), respectively. By comparing the IC_50_ values of HI essential oil with that of the BHT, it was found that the inhibition of lipid peroxidation of HI oil with IC_50_ = 10.48 ± 1.22 mg/mL was comparable with that of BHT (IC_50_ = 7.13 ± 0.54 mg/mL).

### 2.3. Percentage Wound Contraction

After 7-day topical administration, wound healing contraction in rats treated with *H. italicum* essential oil-based ointment and gel were found to be statistically significantly higher when compared to control and vehicle groups. The gel and ointment with incorporated essential oil were found to be the most effective with wound healing contraction of 42.3% and 39.87%, respectively.

The results after 14-day application were similar. The highest percent reduction of wound was in groups treated with essential oil, both in gel and ointment group, with contractions of 84.21% and 82.39%, respectively. The wound healing contraction of the positive control was statistically significantly higher in comparison to the negative control group and the vehicle groups.

**Figure 1:**
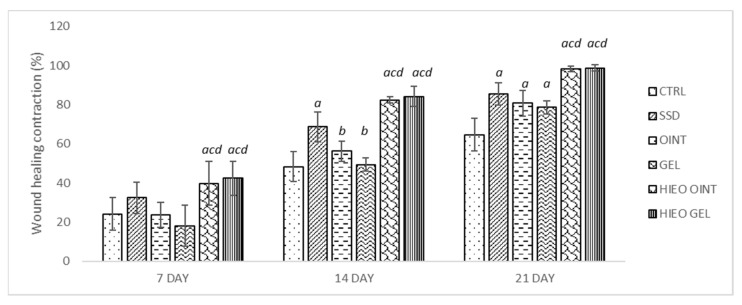
Percentage wound contraction based on treatment. Values are presented as mean ± standard deviation. Values *p* < 0.05 were considered to be statistically significant. *^a^* Statistical significance in relation to CTRL group; *^b^* Statistical significance in relation to SSD group; *^c^* Statistical significance in relation to OINT group; *^d^* Statistical significance in relation to GEL group; CTRL—negative control group; SSD—1% silver sulfadiazine group; OINT—ointment base group; GEL—gel base group; HIEO OINT—*H. italicum* essential oil ointment group; HIEO GEL—*H. italicum* essential oil gel group.

**Figure 2:**
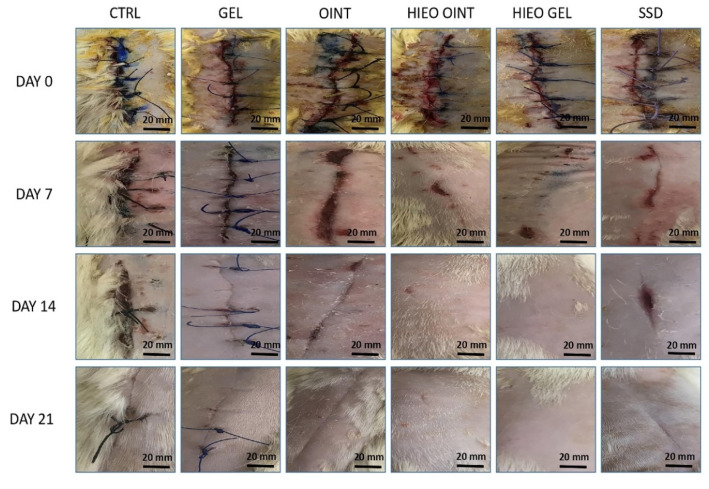
The wound healing effect of *H. italicum* essential oil-based topical formulations on incision wound model on different days (0, 7, 14, 21).

The beneficial effect of *H. italicum* oil from the 7th day was obvious, and at the end of treatment *H. italicum* gel and ointment, with wound contractions of 98.75% and 98.33%, were the most effective among all of the examined groups. On the other hand, animals in the treated groups showed better wound healing contraction than the negative control group (Figure 1 and Figure 2).

### 2.4. Hydroxyproline Content

**Figure 3:**
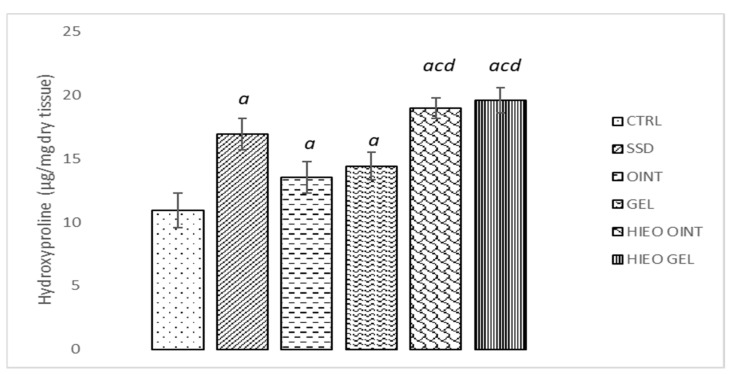
Effects of applied formulations on the hydroxyproline content. Values are expressed as mean ± standard deviation. Values *p* < 0.05 were considered to be statistically significant. *^a^* Statistical significance in relation to CTRL group; *^c^* Statistical significance in relation to OINT group; *^d^* Statistical significance in relation to GEL group.

Animals treated with *H. italicum* oil topically showed the highest hydroxyproline content of granulation tissue. HIEO ointment and HIEO gel showed significant improvement in comparison to negative control and vehicle groups, but did not show a significantly higher amount of hydroxyproline compared to the positive control group. A modest effect on hydroxyproline content was observed in the vehicle groups when compared to the control group (Figure 3).

### 2.5. Redox Status

**Figure 4:**
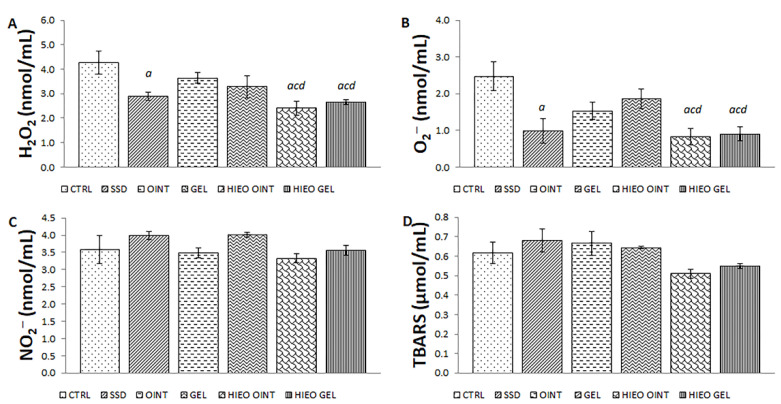
Effects of applied formulations on the pro-oxidative markers: (**A**) H_2_O_2_^−^ (**B**) O_2_^−^; (**C**) NO_2_^−^; (**D**) TBARS. Values are expressed as mean ± standard deviation. Values *p* < 0.05 were considered to be statistically significant. *^a^* Statistical significance in relation to CTRL group; *^c^* Statistical significance in relation to OINT group; *^d^* Statistical significance in relation to GEL group.

Treatment with *H. italicum* essential oil, in both ointment and gel formulations, significantly decreased the level of hydrogen peroxide (H_2_O_2_) release in relation to the negative control ointment and gel groups (Figure 4A). Identically, the level of superoxide anion radical (O_2_^−^) was the least in groups treated with the *H. italicum* essential oil preparations. Significant difference between the animals treated with *H. italicum* preparations was observed compared to control and vehicle groups (Figure 4B). On the other hand, the levels of nitric oxide (NO) and TBARS (thiobarbituric acid-reactive substances) weren’t significantly affected by any of the treatments (Figure 4C,D).

**Figure 5:**
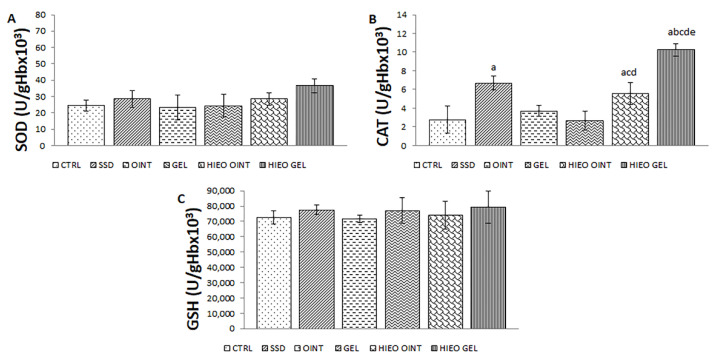
Effects of applied formulations on the parameters of antioxidative defense system: (**A**) SOD; (**B**) CAT; (**C**) GSH. Values are expressed as mean ± standard deviation. Values *p* < 0.05 were considered to be statistically significant. ^a^ Statistical significance in relation to CTRL group; ^b^ Statistical significance in relation to SSD group; ^c^ Statistical significance in relation to OINT group; ^d^ Statistical significance in relation to GEL group; ^e^ Statistical significance in relation to HIEO OINT group.

The levels of non-enzymatic antioxidant GSH (reduced glutathione) and the activities of enzymatic defense systems such as superoxide dismutase (SOD) and catalase (CAT) are depicted in Figure 5. Application of the 0.5% *H. italicum* gel remarkably increased the activity of CAT compared to all other groups. In addition, the HIEO ointment showed significantly higher activity in comparison to both the control and vehicle groups (Figure 5B).

### 2.6. Histologic Analysis

**Figure 6:**
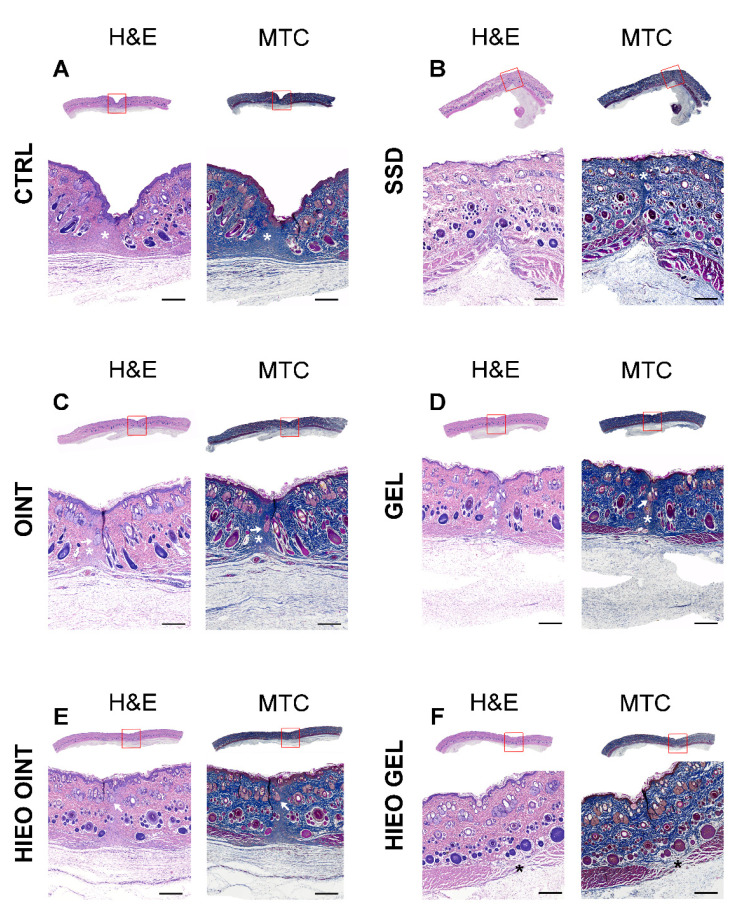
Representative pictures of hematoxylin-eosin (H&E) and Masson Trichrome (MRC) stained tissue sections from CTRL group (**A**), SSD group (**B**), OINT group (**C**), GEL group (**D**), HIEO OINT group (**E**) and HIEO GEL group (**F**). Scale bar, 1 mm.

**Figure 7:**
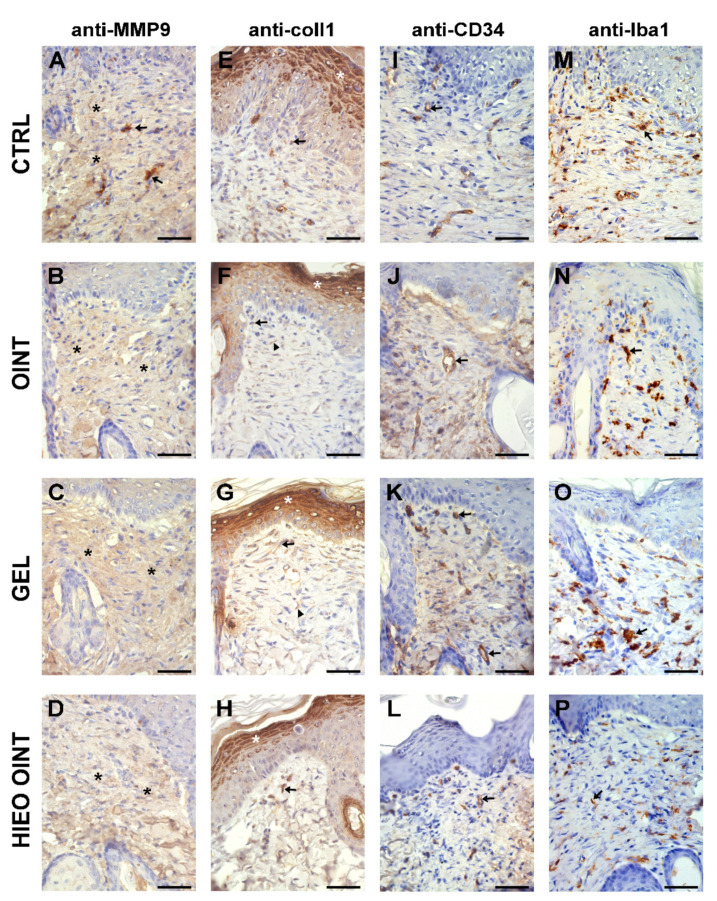
Representative immunohistochemical staining of MMP9, collagen I, CD34 and Iba1 in some animal groups. Scale bar, 50 μm. Representative immunohistochemical staining of MMP9, collagen I, CD34 and Iba1 in CTRL group (**A**,**E**,**I**,**M**), OINT group (**B**,**F**,**J**,**N**), GEL group (**C**,**G**,**K**,**O**) and HIEO OINT group (**D**,**H**,**L**,**P**). Scale bar, 50 μm. * indicate faint staining of the fibroblast cytoplasm in the dermis (black or white asterisk depending on the background); → indicate MMP9-positive cells in (**A**), collagen I-positive fibroblasts in (**E**–**H**), CD34-positive capillary vessels in (**I**–**L**) and Iba1-positive macrophages in (**M**–**P**); ▲ indicate fibers in (**E**–**H**).

Three weeks from the incision, complete re-epithelialization of the epidermal layer was observed in all experimental groups. No significant differences in the thickness and cytological characteristics of the epidermis between the groups were observed. However, the most noticeable changes could be observed in the superficial and primarily in the deep layers of the dermis. The most severe reparative fibrosis (white asterisk) was observed in the negative control group (Figure 6A). Moderate fibrosis (white asterisk), but still without restitution of adnexa, was observed in the positive control group (Figure 6B). The first signs of the return of dermal adnexa, such as sebaceous and sweat glands and hair follicles (white arrow), but still with a presence of fibrosis (white asterisk), were observed in the group’s ointment base and gel base (Figure 6C,D). The use of *H. italicum* essential oil in the ointment and especially the gel formulation led to cytoarchitectonic restitution of the dermis (Figure 6E (white arrow) and Figure 6F). In some individuals from the group treated with *H. italicum* essential oil in the gel formulation, the restitution of adnexal structures was so pronounced that only the underlying muscle defect indicated the incision site (Figure 6F black asterisk). Using MMP9, we noticed a predominantly faint staining of the fibroblast cytoplasm in the dermis of all groups (Figure 7A–D—black asterisk). In the negative control group, we found rare MMP9-positive cells that most likely belonged to macrophages (Figure 7A—black arrows). At 21 days after wounding, in all animal groups, we noticed collagen I-positive fibroblasts (black arrows) and fibers (black arrowheads) (Figure 7E–H). The most pronounced collagen I production was found in the gel group of animals (Figure 7G). Inside the fibrous scar, we identified CD34-positive capillary vessels (black arrows). Looking at the vascularization of the scar, there were no differences among the groups (Figure 7I–L). Although the maturation of the fibrous tissue in the scar was almost finished, we were still able to identify the presence of Iba1-positive macrophages (Figure 7M–P—black arrows). Their predomination was observed in the control group (Figure 7M).

## 3. Discussion

Wounds represent a disorder of the anatomical and cellular continuity of tissue, caused by physical, chemical, thermal, microbial, or immune damage. The wound healing process is a complex and dynamic biological process consisting of integrated cellular and biochemical cascades that lead to the re-establishment of the damaged tissue’s structural and functional integrity [14]. The wound healing process consists of four temporally and spatially overlapping phases: hemostasis, inflammation, proliferation, and remodeling phase [15]. The influence of various factors such as malnutrition, aging, radiation, and diseases such as diabetes, hypertension, and obesity may be associated with delayed wound healing [16]. Research has shown that long-term elevated blood glucose levels, and disorders of its metabolism can have an impact on the stages of wound healing [17].

Since the altered wound healing significantly disturbs patients’ quality of life, there is an urgent need to develop novel therapeutic strategies that can contribute to proper wound healing and exert fewer side effects in comparison to the currently approved pharmacological agents. Previous studies have reported the anti-inflammatory and antioxidant effects of *H. italicum* essential oil, and therefore we hypothesized that essential oil prepared from this plant species could be used to accelerate the wound healing process under diabetic conditions. In that sense, we investigated the effects of ointment and gel based on *H. italicum* essential oil in wound healing, with a special focus on revealing the impact of these topical preparations on redox status and structural tissue alterations.

In the first part of our research, we aimed to reveal the chemical composition and antioxidant capacity of the *H. italicum* essential oil. The most represented compounds in *H. italicum* essential oil are the sesquiterpene hydrocarbons, with a predominance of γ-curcumene and β-selinene, as well as monoterpenes, with α-pinene and neryl acetate as major components. The composition of the present oil is in accordance with previously reported literature data. A-pinene and γ-curcumene are the main ingredients of *H. italicum* essential oil from the Adriatic coast [18,19,20,21], while oil from Tuscany mainly contained α-pinene and neryl acetate [22]. The dominant component in oil from Sardinia was also γ-curcumene, while sesquiterpene hydrocarbons were the most represented chemical class in Crete oil [23]. On the other hand, oil from the Greek island of Amorgos was dominated by geraniol [24]. Therefore, the chemical composition of immortelle essential oil demonstrates intraspecific differences in response to environmental factors such as geographic origin, vegetation cycle, soil properties, altitude, and climatic conditions [21]. The variability of the bioactive compounds, and sometimes synergistic effects between them have a strong influence on their biological activity [21].

The investigation of the in vitro antioxidant potential of *H. italicum* oil included the assessment of the ability of compounds present in essential oil to free-radical-scavenging capacity, such as DPPH·, NO, and OH. In addition, the ability of the examined oil to protect the integrity of biological membranes containing lipids was evaluated through the determination of lipid peroxidation inhibition potential. Immortelle essential oil exhibited an IC50 value of 4.45 mg/mL for inhibiting DPPH radicals, which is poor compared to the 0.69 mg/mL exhibited by standard propyl gallate. In line with our findings, an investigation by Kladar et al. found a 3ȕ lower DPPH scavenging capacity for *H. italicum* oil than for propyl gallate. The previous study aimed to compare the chemical composition and antioxidant activity of essential oil originating from Bosnia and France and concluded that the chemical composition was in direct line with the antioxidant activity. French oil was found to be rich in oxygenated monoterpenes, while the oil obtained from Bosnian *H. italicum* was rich in sesquiterpene hydrocarbons, and consequently essential oil from France exhibited higher antioxidant activity [25]. A potential explanation for the insufficiently potent in vitro antioxidant activity might lie in the fact that essential oils and their pure compounds are not soluble in polar solvents, and therefore they could not show powerful antioxidant activity [26].

The important factors in the wound healing process are contraction and epithelialization [2], and therefore, monitoring these phases is of crucial importance in assessing the wound healing potential of *H. italicum* essential oil. The results of the present study showed that the contraction values were higher in groups treated with topical preparation based on *H. italicum* essential oil, thus indicating shorter time for re-epithelialization in comparison to other groups. The wound healing potential may be due to the presence of terpenes with confirmed astringent and antimicrobial potential, which help to improve wound contraction and facilitate epithelialization [27,28]. The former investigation demonstrated that *H. italicum* essential oil possesses strong and significant anti-proliferative effects in a human dermal fibroblast system. The essential oil primarily inhibited tissue remodeling-related proteins, such as collagen I and III, suggesting a promising wound healing property [29]. Important mediators implicated in the regulation of angiogenesis and tissue remodeling during diabetic wound healing are metalloproteinases [30]. Metalloproteinases, such as MMP-9, can be released rapidly and they are activated at the initial stages of wound healing. The higher MMP-9 activity is linked to a reduction in fibroblast proliferation and activity, which leads to the impaired healing seen in diabetic wounds. On the other hand, low levels of MMPs, such as MMP-1 and MMP-9 found in the epithelialized wound are associated with solving the pathologic state [30,31]. Thus, the healed areas of the wound lose active MMP-1 and MMP-9 expression and this in correlation with our study in which complete re-epithelialization was observed on day 21 after wounding. The density of capillaries in scar tissue can be expressed through the expression of CD34. CD34, as a marker in tissue, reflects the healing process by increasing vascular endothelial cell motility, which is beneficial for endothelial repair and vascular reconstruction [32]. It is probable that the period of three weeks after the surgical incision and complete re-epithelialization contributed to the fact that there were no differences between the groups in the vascularization of the scar. Treatment with topical formulations significantly inhibited the subsequent infiltration of Iba1-positive cells (macrophages) compared to the control group, where their predomination was noticed.

One of the most abundant components in *H. italicum* essential oil, α-pinen, also promotes the wound healing process by accelerating wound closure, generating scars with effective tensile strength, and contributing to collagen deposition. A slight increase in collagen deposition was noticed in the wound treated with α-pinen, which is very important, because the wound repair process depends on the biosynthesis, deposition, and maturation of collagen. In addition, collagen deposits in the wounds provide resistance to the tension of the scars formed [33].

Taking into consideration that collagen is the major component strengthening and supporting extracellular tissue, we aimed to indirectly determine its content in skin tissue by determining hydroxyproline content [34]. Hydroxyproline permits the sharp twisting of the collagen helix, and high concentrations direct cell proliferation, as well as the synthesis, localization and maturation of collagen, all of which are in direct connection with the rate of wound healing [35]. A significant increase in hydroxyproline content in groups treated with *H. italicum* clearly suggests that the essential oil of *H. italicum* enhances collagen synthesis and deposition, which provides strength and tissue integrity, thereby playing a vital role in tissue epithelialization and homeostasis [36]. Thus, the essential oil of *H. italicum* is among the treatments of choice in the immediate postoperative therapeutic arsenal, by offering real efficiency and good tolerance through reduction of local inflammation, edema, hematomas, and bruises [37].

Oxidative stress represents one of the crucial factors contributing to disturbed wound healing processes, thus indicating that plant-based products with antioxidant activity may be a useful tool for skin repair [38]. Since previous studies have confirmed the in vivo antioxidant effects of *H. italicum*, we hypothesized that chronic application of ointment and gel based on its essential oil might significantly accelerate wound healing. The special focus of the current research was to follow the impact on systemic redox status of treatment with topical preparations based on *H. italicum*. Our findings suggest that rats treated with both *H. italicum* ointment and gel showed a significant increase in the activity of CAT with a decrease in NO and H_2_O_2_ levels compared to controls. Reduction in pro-oxidant levels and enhancement in antioxidant enzyme activity achieved by *H. italicum* formulations indicate that alleviation of oxidative stress is one of the mechanisms responsible for wound healing effects.

To thoroughly assess the role of *H. italicum* essential oil-based topical formulations in wound healing, we followed the structural skin alterations after the treatment. Histopathological analysis confirmed that the topical formulations of *H. italicum* essential oil were beneficial in wound therapy. Topical application of the formulation based on *H. italicum* essential oil led to cytoarchitectonic restitution of the dermis, especially pronounced in gel group with only the underlying muscle defect indicating the incision site. Therefore, the essential oil can be administered incorporated into either a gel or an ointment, as both are effective at promoting healing. The potentially better effects of gel formulation may be explained by the fact that gel coagulates blood within a very short period of time. Other advantages of gel as a dosage form involve its easy application and washability. On the other hand, ointments are sticky, oily, and not soothing to apply to wounds, and their actions at controlling bleeding are very slow [39].

The results of the wound healing effects in our research legitimate the traditional data of the pro-healing effect of *H. italicum*, since in both formulations (gel and ointment), *H. italicum* exhibited a significant wound repairing effect in the incision wound model, validated by a significant acceleration in the wound contraction rate and content of hydroxyproline. These positive wound healing effects were supported by biochemical estimation and histopathological studies.

## 4. Materials and Methods

### 4.1. Test Compounds

The essential oil of *H. italicum,* Asteraceae, obtained by hydrodistillation from flowering parts of *H. italicum* was purchased from “Alekpharm”, Belgrade, Serbia. The essential oil is 100% pure.

### 4.2. Phytochemical Analysis of H. italicum Essential Oil

To identify the qualitative and quantitative composition of the *H. italicum* essential oil, analysis was carried out on the HP-5MS capillary column (30 m × 0.25 mm; film thickness 0.25 μm) on Agilent 6890B GC-FID instrument coupled to Agilent 5977 MSD according to a previously published study [40].

### 4.3. Antioxidant Potential Evaluation

Five in vitro assays were used for evaluation of the antioxidant activity of *H. italicum* essential oil. Spectrophotometric methods were used to evaluate the essential oil’s potential to neutralize DPPH radicals, NO and OH radicals. Additionally, LP inhibition potential of essential oil was tested and FRAP test was performed in the current study. Ascorbic acid (AA), propyl gallate (PG), and tert-butylated hydroxytoluene (BHT) were used as standard antioxidants. In all test systems, four duplicates of each sample were recorded [41,42,43,44]. All results, except those obtained in FRAP test, are presented as the IC_50_ value, which represent the concentrations of the essential oil and positive controls that caused 50% of neutralization, determined by linear regression analysis. In the FRAP test results are presented as AA equivalents.

### 4.4. Preparation of the Test Formulations

The ointment of *H. italicum* essential oil was prepared according to Valizadeh et al.’s protocol [45]. The ointment was prepared by mixing 0.5 g *H. italicum* essential oil with Eucerin ointment base until a homogenized sample was obtained. The Eucerin base contained a mixture of cholesterol (5 g), lanolin (15 g), paraffinum liquidum (15 g) and vaselinum album (65 g).

For the preparation of a simple gel base, carbomer, propylene glycol, triethanolamine solution and water were used. The carbomer was mixed with water until dispersion is formed. The dispersion was stirred with propylene glycol, triethanolamine solution was added, and the mixture was allowed to swell. The 0.5% *w*/*w* gel of essential oil was prepared by incorporating 0.5 g oil into a 99.5 g carbomer mucilage gel.

The choice of 0.5% as a concentration of essential oil in gel and ointment was based on literature data and relevant recommendations. Studies suggest that a dose of 0.5% appears to be suitable for topical application, while increasing the dose is not recommended due to the potential for skin irritation [46].

### 4.5. In Vivo Wound Healing Studies

#### 4.5.1. Animals

Forty-eight male Wistar albino rats (280–350 g) obtained from the Military Medical Academy, Belgrade, Serbia, were used for the in vivo wound healing experimental study. The animals were housed in clean cages on an artificial 12-h light–dark cycle (8:00 a.m–8:00 p.m.) at a temperature of 22 ± 2 °C. The rats had free access to water and food ad libitum. This investigation was conducted in the Laboratory for cardiovascular physiology and Laboratory for pharmaceutical technology of the Faculty of Medical Sciences, University of Kragujevac, Kragujevac, Serbia. The Ethics Committee of the Faculty of Medical Sciences (Number 01-6292, 31 July 2020) approved the experimental protocol, and all procedures were performed following EU Directive for Protection of the Vertebrate Animals used for Experimental and other Scientific Purposes 86/609/EES and the principles of ethics.

#### 4.5.2. Induction of Diabetes

Diabetes was induced with a single intraperitoneal injection of streptozotocin (50 mg/kg) dissolved in 1 mL of 0.05 M freshly prepared citrate buffer solution (pH 4.5) after 12 h starvation [47,48]. Three days after streptozotocin injection following overnight fasting, diabetes was confirmed by measuring tail veinous blood glucose level using a portable glucometer. Animals with blood glucose levels above 11.1 mmol/L were considered to have diabetes and were chosen as wound models.

#### 4.5.3. Incision Wound Model

One week after confirmation of diabetes, incision wounds were made [49]. To create incision wounds, rats were anesthetized using a mixture of ketamine (5 mg/kg) and xylazine (10 mg/kg) intraperitoneally. The dorsal backs were shaved and disinfected with 70% ethanol. Then, 6-cm-long para-vertebral incisions were created on the dorsal side and closed with sutures at distances of 1 cm [50].

#### 4.5.4. Experimental Animals

Immediately after the wounds were created, rats were placed in separate cages and the treatments were started by daily application the test formulations. Depending on the applied formulation, the rats were divided randomly into six groups (8 per group) as follows:

1. Negative control, the wound was left without intervention
2. Positive control (the wound was treated with 1% silver sulfadiazine)
3. Ointment base (the wound was treated with Eucerin base ointment)
4. Gel base (the wound was treated with Carbomer mucilago gel)
5. HIEO ointment (the wound was treated with the 0.5% *H. italicum* essential oil ointment).
6. HIEO gel (the wound was treated with the 0.5% *H. italicum* essential oil gel).

The test formulations were topically applied, in the amount of 0.5 g by a sterile cotton swab, on the dorsal wounds in each group once daily for 21 days [3,51]. The sutures were removed on the 21st day post wounding and the animals were sacrificed. Blood was collected for determination of systemic redox status and tissues were taken for histopathological analysis and estimation of hydroxyproline.

#### 4.5.5. Estimation of Wound Contraction and Epithelialization Period

After their creation, the wounds were photographed and the lengths of the incisions were recording using graph paper and Image J software measured on the 0th, 7th, 14th and 21st post-wound days. Wound healing ratio was represented as percentage of contraction/closure and was calculated for each animal using the following formula [52]:

% Wc = (Wa 0−Wa N) × 100/Wa 0(1)

where % Wc—% wound contraction; Wa 0—Wound area on day 0; Wa N—Wound area on particular day.

### 4.6. Biochemical Analysis

#### 4.6.1. Hydroxyproline Estimation

Hydroxyproline index was measured to determine the total collagen content in the tissues. Tissue samples were completely dried at 60–70 °C for 12–18 h and allowed to hydrolyze in 6 M HCl (1 mL HCl/10 mg dry tissue) at 105 °C for 4 h. The hydrolysate was centrifuged at 3000 rpm for 15 min and 1 mL of supernatant was mixed with chloramine-T. After 20 min, 1 mL of Ehrlich’s reagent was added, and the mixture was incubated for 30 min at 60 °C. Absorbance was recorded at 558 nm via spectrophotometer and hydroxyproline content estimated as μg/mg dry tissue weight after plotting the absorbance against a pure hydroxyproline calibration curve [53].

#### 4.6.2. Evaluation of Systemic Redox State

Blood samples were collected from jugular vein in the moment of sacrificing animals. The concentration of pro-oxidative markers was evaluated spectrophotometrically by NO_2_^−^, H_2_O_2_, O_2_^−^, and index of lipid peroxidation measured as a TBARS in plasma samples.

In the lysate of erythrocytes, parameters of the antioxidative defense system such as level of GSH, and activities of CAT and SOD were determined.

##### Determination of the Index of Lipid Peroxidation Measured as TBARS

Index of lipid peroxidation was estimated by measuring TBARS using 1% thiobarbituric acid (TBA) in 0.05 NaOH, incubated with plasma at 100 °C for 15 min and measured spectrophotometrically at 530 nm [54].

##### Nitrite Determination (NO_2_^−^)

The level of NO_2_^−^ was measured spectrophotometrically as an index of nitric oxide (NO) production with Griess’s reagent [54]. A total of 0.2 mL of plasma was precipitated with 100 µL of 3 N perchloric acid and 0.4 mL 20 mM ethylenediaminetetraacetic acid, put on ice for 15 min, and centrifuged at 6000 rpm for 15 min. After that, 220 μL of potassium carbonate was added in sludge and 200 μL of the sample was mixed with 250 μL Griess’s reagent and 125 μL of buffer for NO. After 15-min incubation, nitrite levels were measured at a wavelength of 550 nm.

##### Superoxide Anion Radical Determination (O_2_^−^)

The level of O_2_^−^ was measured after reaction of nitro blue tetrazolium in TRIS (tris(hydroxymethyl)aminomethane) buffer (assay mixture) with the plasma at 530 nm [54].

##### Hydrogen Peroxide Determination (H_2_O_2_)

The measurement of the level of H_2_O_2_ was based on oxidation of Phenol Red by hydrogen peroxide, in a reaction catalyzed by horseradish peroxidase (prepared ex tempore) at 610 nm [54].

##### Determination of Reduced Glutathione (GSH)

The level of GSH was determined on the basis of GSH oxidation with 5.5-dithio-bis-6.2-nitrobenzoic acid and detection was performed at a wavelength of 420 nm [54].

##### Determination of Catalase (CAT)

CAT activity was determined according to Aebi. Lysates were diluted with distilled water (1:7 *v*/*v*) and treated with chloroform–ethanol (0.6:1 *v*/*v*). Then, 50 μL buffer for CAT, 100 μL of prepared sample and 1 mL 10 mM H_2_O_2_ were used for the determination. Detection was performed at 360 nm [54].

##### Determination of Superoxide Dismutase (SOD)

The epinephrine method was used to determine SOD activity. A total of 100 μL of epinephrine was added in the mixture of 100 μL of lysate and 1 mL of carbonate buffer. Detection was performed at a wavelength of 470 nm [54].

### 4.7. Histologic Analysis

After sacrifice, samples of the rat’s skin were fixed at 10% buffered formalin solution at 4 °C for 24 h for histological examination. After fixation, tissue sections were dehydrated in increasing concentrations of isopropyl alcohol (70%, 80%, 96% and 100%), molded into the paraffin and cut on a rotating microtome (Leica, Germany) to a thickness of 5 μm. The tissue was stained with hematoxylin-eosin (H&E) and Masson’s trichrome (MTC) and the following immunohistochemical markers: rabbit anti-MMP9 in a 1:25 dilution (Lab Vision; Thermo Scientific, Rockford, IL, USA); rabbit anti-collagen I in a 1:300 dilution (Abcam; Cambridge, UK); rabbit anti-CD34 in a 1:3500 dilution (Abcam; Cambridge, UK) and rabbit anti-Iba1 in a 1:8000 dilution (Abcam; Cambridge, UK). For visualization, we used Mouse and Rabbit Specific HRP/DAB (ABC) Detection IHC kit (Abcam; Cambridge, UK). Retrieval reaction for slides stained with anti-collagen I and anti-MMP9 was TRIS base buffer (pH 8.2), and for anti-CD34 and anti-Iba1 was citrate buffer (pH 6.0). All the antibodies were applied for 60 min at room temperature. Counterstain for immunohistochemistry was done with Mayer’s hematoxylin. Finally, slides were mounted within DPX medium (Sigma-Aldrich, Darmstadt, Germany) and coverslipped. Histological slides were analyzed under a Leica DMLB 100T professional biological microscope (Leica, Wetzlar, Germany) and scanned on a digital microscope VisionTek^®^ (Sakura, Tokyo, Japan).

### 4.8. Statistical Analysis

IBM SPSS Statistics 20.0 Desktop for Windows was used for statistical analysis. All data were expressed as mean ± standard deviation. The Shapiro–Wilk test was used to assess the distribution of data. For the normal distribution, the data were analyzed by one-way analysis of variance (ANOVA) followed by Tukey’s post hoc test for multiple comparisons. When the distribution was different from than normal Kruskal–Wallis test was used for the comparison between groups. A *p*-value < 0.05 was considered to be statistically significant.

## 5. Conclusions

The results of the present study verify the fact that gel and ointment prepared from *H. italicum* essential oil could be a topical herbal remedy for improved healing wounds in a diabetes rat model. In addition, clinical trials must be implemented to confirm that the promising activities of the *H. italicum* essential oil formulation can be safely and effectively implemented with clinical utility and to fully confirm its recognized use in traditional medicine.

## Figures and Tables

**Table 1:** Chemical composition of the *H. italicum* essential oil.

Peack No.	Compound	RI	%
**Monoterpene Hydrocarbons**			18.52
1	α-Pinene	937	12.38
2	Camphene	952	0.43
3	β-Pinene	978	0.44
4	β-Myrcene	991	0.03
5	δ-2-Carene	1001	0.13
6	α-Phellandrene	1005	0.06
7	δ 3-carene	1011	0.05
8	α-Terpinene	1017	0.25
10	Limonene	1030	3.74
12	cis-β-Ocimene	1037	0.43
13	γ-Terpinene	1060	0.45
14	Terpinolene	1088	0.13
**Aromatic Monoterpene Hydrocarbons**			0.37
9	p-Cymene	1025	0.37
**Oxigenated Monoterpenes**			15.56
11	1,8-Cineole	1032	0.23
16	Linalool	1099	0.76
17	Fenchol	1113	0.13
19	endo-Borneol	1167	0.06
20	Terpinen-4-ol	1177	0.23
21	α-Terpineol	1189	0.28
22	Nerol	1228	0.74
23	Geraniol	1253	0.17
23	Neryl acetate	1364	12.96
**Sesquiterpene Hydrocarbons**			59.62
24	α-Cubebene	1351	2.37
25	Ylangene	1372	0.17
26	α-Copaene	1376	0.34
27	β-Cubenene	1388	0.97
28	Italicene	1403	3.58
29	cis-α-Bergamotene	1415	0.93
30	*trans*-β-Caryophyllene	1419	4.89
31	Cedrene	1422	0.45
32	*trans*-α-Bergamotene	1435	0.88
33	Aromandendrene	1441	1.06
34	Humulene	1454	0.48
35	Alloaromadendrene	1461	2.66
36	Acoradiene	1471	0.44
28	ar-Curcumene	1480	1.07
29	γ-Curcumene	1483	14.07
40	β-Selinene	1486	11.27
41	α-Selinene	1494	7.27
42	δ-Selinene	1497	3.36
43	α-Muurolene	1499	0.95
44	δ-Cadinene	1524	2.41
**Oxigenated Sesquiterpenes**			0.77
45	Caryophyllene oxide	1581	0.77
46	*trans*-Farnesol	1725	0.26
**Aliphatic compounds**			0.34
21	Dodecane	1201	0.34
**Other**			3.19
15	Butyl angelate	1091	0.29
18	2-Methylbutyl angelate	1146	1.25
37	Geranyl propionate	1475	1.65
Total of identified compounds			98.37

RI—Retention indices relative to C9–C24 n-alkanes on the HP 5MS column.

**Table 2:** Antioxidant potential of *H. italicum* essential oil and positive control substances.

Samples	Assay				
	DPPH IC_50_	OH IC_50_	NO IC_50_	LP IC_50_	FRAP
	(µg/mL)				(mg AAE/mL HIEO)
HIEO	4.45 ± 0.44	13.33 ± 1.11	n.d.	10.48 ± 1.22	0.03 ± 0.00
AA	/	2.03 ± 0.39	/	/	/
PG	0.69 ± 0.03	9.01 ± 0.48	/	/	/
BHT	/	0.03 ± 0.01	/	7.13 ± 0.54	/

Data presented as means ± standard deviations. HIEO—*Helichrysum italicum* essential oil; AAE—ascorbic acid equivalents; AA—ascorbic acid, PG—propyl gallate; BHT—tert-butylated hydroxytoluene. DPPH—2,2-diphenyl-1-picrylhydrazyl; OH—hydroxyl ion; NO—nitric oxide; LP—lipid peroxidation; FRAP—ferric reduction antioxidant potential.

## Data Availability

Data is contained within the article.
